# Supplementary material for: Surface Treatment of Cu:NiOx Hole-Transporting Layer Using β-Alanine for Hysteresis-Free and Thermally Stable Inverted Perovskite Solar Cells
Source: Nanomaterials (Basel). 2020 Oct 1;10(10):1961. doi: 10.3390/nano10101961 (PMC7599611; doi:10.3390/nano10101961)
Supplement: Supplementary file 1 [file nanomaterials-10-01961-s001.pdf]

nanomaterials

MDPI

*Supplementary Materials*

**Fedros Galatopoulos, Ioannis T. Papadas, Apostolos Ioakeimidis, Polyvios Eleftheriou and Stelios A. Choulis ***

Molecular Electronics and Photonics Research Unit, Department of Mechanical Engineering and Materials Science and Engineering, Cyprus University of Technology, 3603 Limassol, Cyprus; fedros.galatopoulos@cut.ac.cy (F.G.); ioannis.papadas@cut.ac.cy (I.T.P.); a.ioakeimidis@cut.ac.cy (A.I.); polyvios.eleftheriou@cut.ac.cy (P.E.)

* Correspondence: stelios.choulis@cut.ac.cy

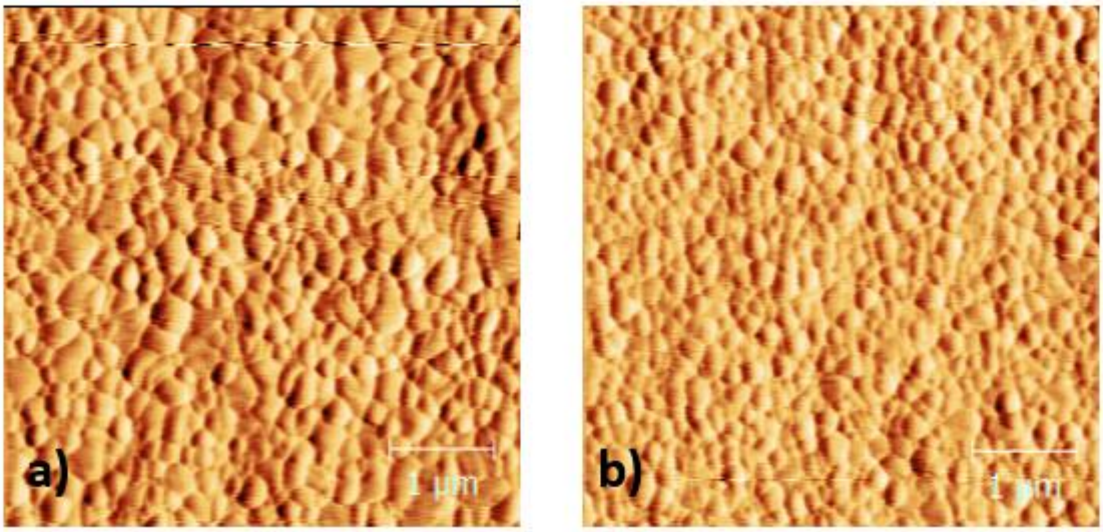


**Figure S1.** AFM measurements in phase contrast of (**a**) ITO/Cu:NiOx/Pvsk, (**b**) ITO/Cu:NiOx/βalanine/Pvsk.

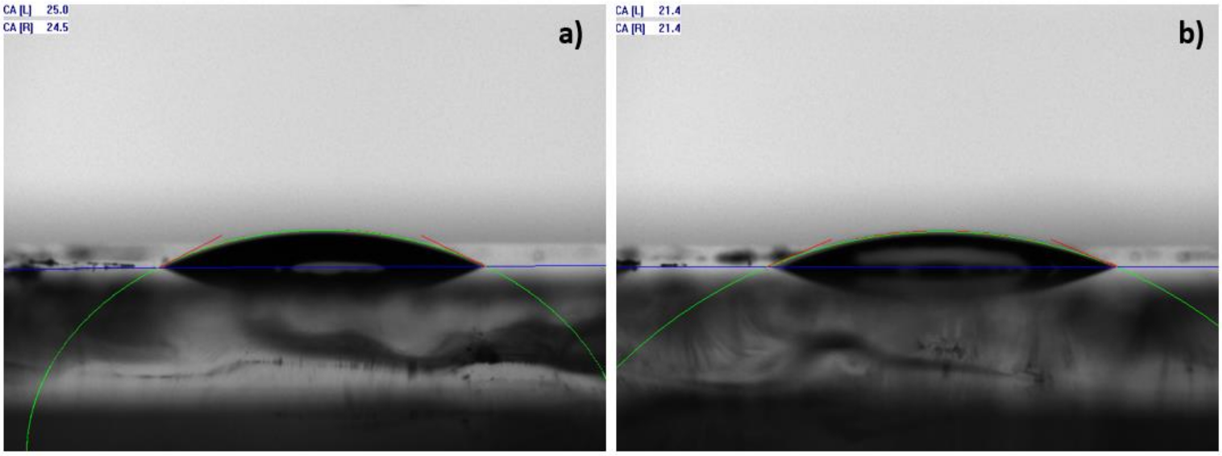


**Figure S2.** Contact angle film measurements of (**a**) Cu:NiOx, (**b**) Cu:NiOx/alanine using γ-butyrolactone:DMSO (7:3) for the perovskite solvent.

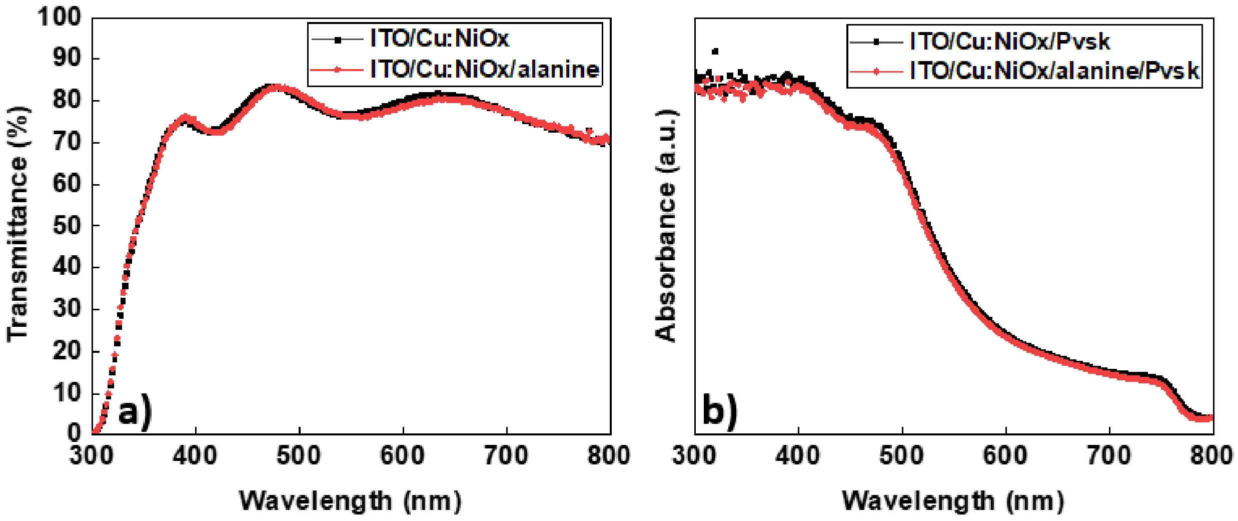


**Figure S3.** (**a**) Transmittance spectra of ITO/Cu:NiOx , ITO/Cu:NiOx/alanine and (**b**) Absorbance spectra ITO/Cu:NiOx/Pvsk, ITO/Cu:NiOx/β-alanine/Pvsk.

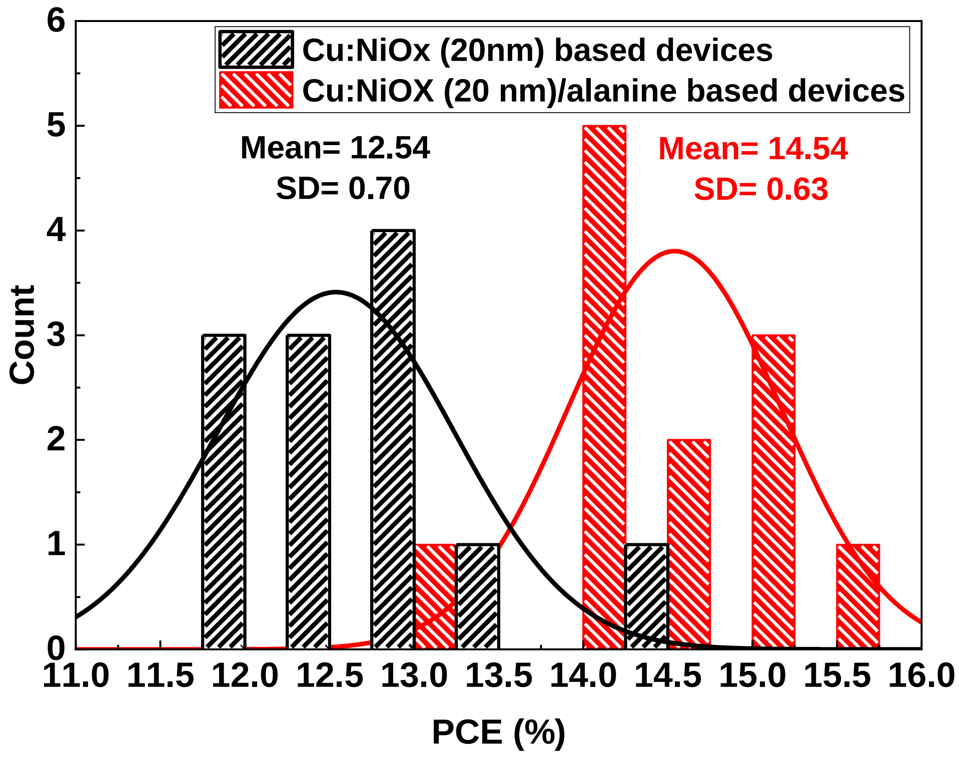


**Figure 4.** Average PCE and SD for the optimized devices based on Cu:NiOx (20 nm).

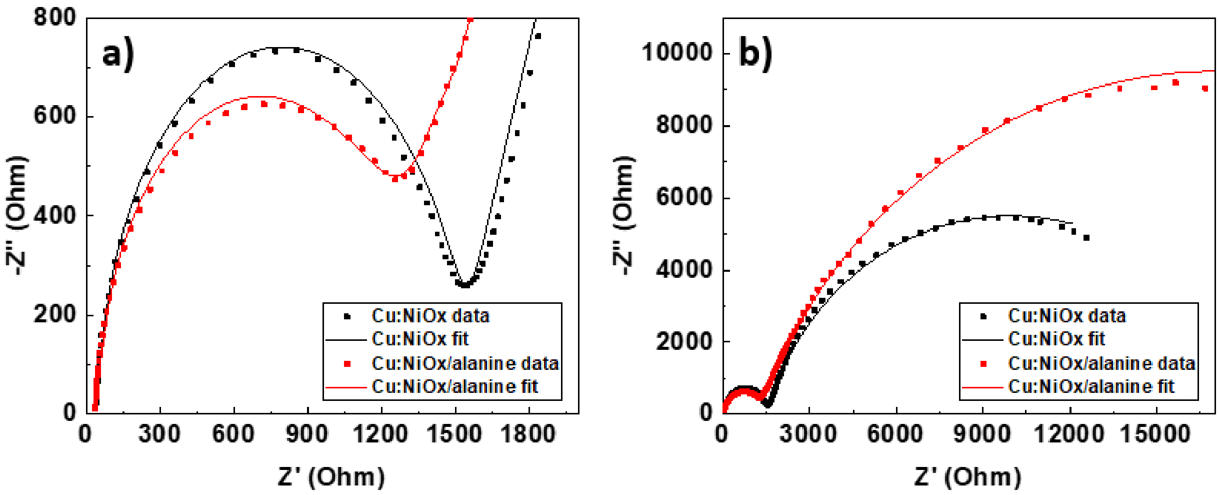


**Figure S5.** Nyquist data and fitting for (**a**) $R_{tr}$ and (**b**) $R_{rec.}$

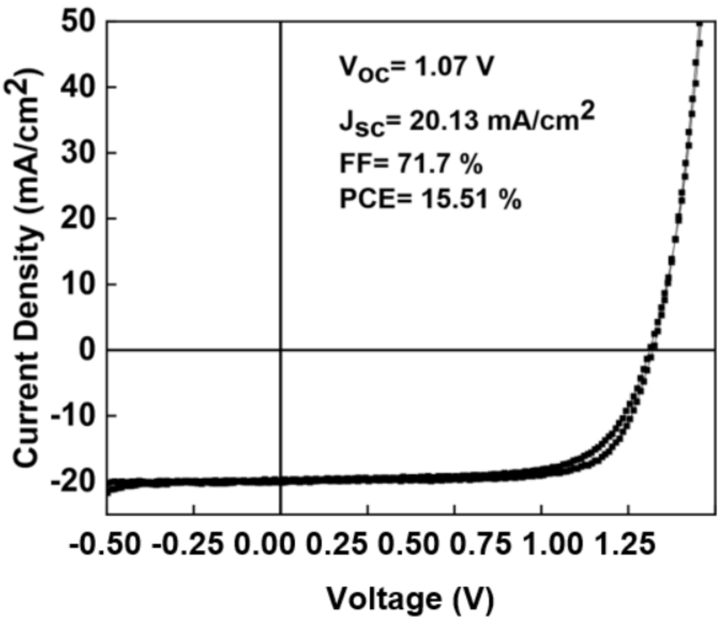


**Figure S6.** J/V characteristic of optimized ITO/Cu:NiOx/β-alanine/Pvsk/$PC_{70}BM$/BCP/Cu.
